# Prevalence and correlates of cancer survivors’ supportive care needs 6 months after diagnosis: a population-based cross-sectional study

**DOI:** 10.1186/1471-2407-12-150

**Published:** 2012-04-18

**Authors:** Allison W Boyes, Afaf Girgis, Catherine D’Este, Alison C Zucca

**Affiliations:** 1 Priority Research Centre for Health Behaviour, University of Newcastle & Hunter Medical Research Institute, Newcastle, Australia; 2 Ingham Institute for Applied Medical Research, University of New South Wales, Liverpool, Australia; 3 Centre for Clinical Epidemiology and Biostatistics, University of Newcastle, Newcastle, Australia

## Abstract

**Background:**

An understanding of the nature and magnitude of the impact of cancer is critical to planning how best to deliver supportive care to the growing population of cancer survivors whose need for care may span many years. This study aimed to describe the prevalence of and factors associated with moderate to high level unmet supportive care needs among adult cancer survivors six months after diagnosis.

**Methods:**

A population-based sample of adult cancer survivors diagnosed with one of the eight most incident cancers in Australia was recruited from two state-based cancer registries. Data for 1323 survivors were obtained by self-report questionnaire and linkage with cancer registry data. Unmet needs were assessed by the 34-item Supportive Care Needs Survey (SCNS-SF34). The data were examined using chi-square and multiple logistic regression analyses.

**Results:**

A total of 444 (37%) survivors reported at least one ‘moderate to high’ level unmet need and 496 (42%) reported ‘no need’ for help. Moderate to high level unmet needs were most commonly reported in the psychological (25%) and physical aspects of daily living (20%) domains. The five most frequently endorsed items of moderate to high unmet need were concerns about the worries of those close to them (15%), fears about the cancer spreading (14%), not being able to do the things they used to do (13%), uncertainty about the future (13%) and lack of energy/tiredness (12%). Survivors’ psychological characteristics were the strongest indicators of unmet need, particularly caseness for anxious preoccupation coping which was associated (OR = 2.2-5.9) with unmet need for help across all domains.

**Conclusions:**

Unmet supportive care needs are prevalent among a subgroup of survivors transitioning from active treatment to survivorship, although lower than previously reported. In addition to coping support, valuable insight about how to prevent or address survivors’ unmet needs could be gained by examining the substantial proportion of survivors who report no unmet needs.

## Background

Cancer is increasingly recognised as a chronic illness, with the number of people living with a history of the disease expected to triple to 75 million people worldwide in 2030 [1]. While most survivors adjust well over time [2], a minority are at risk of adverse physical [3,4], psychological [5,6] and social [7,8] effects which may emerge soon after diagnosis and treatment, or in the ensuing years. Detailed knowledge about the issues faced by survivors, their care and support needs, and the extent to which these are met by current services is critical to guiding where to focus limited healthcare resources in order to deliver care that is responsive to the needs of the growing population of cancer survivors.

There are a number of different approaches for more fully understanding survivors’ cancer experiences and quantifying their outcomes including assessment of quality of life, satisfaction with health care, and needs assessment [9,10]. Needs assessment not only identifies needs and their importance as perceived by the survivor, but also the extent to which they are met [10]. The key strength of this approach is that it enables resources to be focused on the issues that survivors have expressed they want addressed in order to achieve optimal wellbeing.

Increasing interest in the application of needs assessment to cancer care has resulted in the development of a number of valid and reliable cancer-specific tools assessing a comprehensive range of needs [9,11] and a growing literature describing their administration across a variety of settings, stages in the cancer journey, and populations. A recent systematic review found that while the prevalence of unmet need among cancer survivors varied from 30% to 50% across studies, it is typically highest in the psychological, health information, and physical aspects of daily living domains [12]. While evidence about the factors that influence survivors’ unmet needs is inconsistent, a number of studies have found that those who are not in remission [13-15], are psychologically distressed [14,16-18] and geographically isolated [15,19] are more likely to report unmet needs. However, the literature is plagued by a lack of consistency in the methods used to measure, classify and report unmet needs, making it difficult to compare between studies and to generalise findings [12].

The seminal publication, *From Cancer Patient to Cancer Survivor: Lost in Transition*[20], focused the attention of the cancer control community on the survivorship stage of the cancer trajectory with a series of recommendations to accelerate progress in this area, including the need for large-scale studies using valid and reliable measures with diverse cancer populations to be conducted as a priority. Furthermore, a recent review [21] identified unmet supportive care needs as one of four main gaps in knowledge about the problems faced by adult cancer survivors. To guide care planning and help inform future health service delivery, the current study aimed to (1) describe the prevalence of adult cancer survivors’ supportive care needs, overall and by cancer type, at six months post-diagnosis; (2) identify the most prevalent items of moderate to high level unmet need and (3) identify the individual, disease, health behaviour, psychological and social factors associated with survivors reporting moderate to high level unmet psychological, health systems and information, physical and daily living, patient care and support, and sexuality needs.

## Methods

This paper is based on Time 1 (T1) data collected at six months post-diagnosis from survivors participating in the population-based longitudinal *Cancer Survival Study* (CSS). The study protocol and aspects of the study findings have been reported in detail elsewhere [22,23]. While the term cancer ‘survivor’ has varied definitions [24], this study considers ‘survivor’ to encompass anyone diagnosed with cancer, from the time of diagnosis to the end of life [25]. This paper focuses on survivors in the late treatment to early survivorship phase of the cancer continuum.

### Participants & procedures

The sample was recruited from new notifications to the two largest state-based cancer registries in Australia which together account for 60% of all new cancer cases diagnosed [26]. Eligibility was restricted to those who were (1) diagnosed in the previous six months with their first primary cancer of one of the top eight incident cancer types in Australia (prostate, colorectal, female breast, lung, melanoma, non-Hodgkin’s lymphoma, leukaemia, head & neck); (2) aged between 18 and 80 years and living in the state of New South Wales (NSW) or Victoria (VIC) at diagnosis; (3) considered by their physician to be aware of their diagnosis, physically and mentally capable of participating in the study, and sufficiently proficient in English to complete a questionnaire; and (4) alive.

The recruitment and survey methodology have been described in detail previously [22]. Briefly, eligible potential participants whose physician had given active (NSW) or passive (VIC) consent for them to be contacted about the study received a mailed package from the registries. Eligible survivors who agreed to the registries passing on their contact details to the researchers were sent a self-administered questionnaire to complete. Non-responders were sent a reminder questionnaire three weeks later and received a reminder phone call after a further three weeks. A three week interval was used to allow adequate time for survivors to receive, respond to and return the mailed questionnaire prior to receiving a reminder. Return of the questionnaire to the research team indicated voluntary consent to participate. The Human Research Ethics Committees of the University of Newcastle (H-199-1101), Cancer Institute NSW and Cancer Council Victoria approved the study.

### Measures

Data were collected by self-administered questionnaire with additional clinical information obtained from the Cancer Registries for each participant.

### Outcome measure

*Supportive care needs* were measured by the 34-item Supportive Care Needs Survey (SCNS-SF34) which assesses cancer-specific perceived needs across five factor analytically derived domains: psychological (10 items), health systems and information (11 items), patient care and support (5 items), physical and daily living (5 items), and sexuality (3 items) [27]. For each item, respondents indicate their level of need for help over the last month as a result of having cancer on a five point Likert scale with the following response options: 1 = no need, not applicable; 2 = no need, satisfied; 3 = low need; 4 = moderate need; and 5 = high need. For each domain, survivors were categorised as having a ‘moderate to high’ level of need if they selected response options 4 or 5 to at least one item in the domain or ‘no to low’ need if they selected response options 1, 2 or 3 to all items in the domain [28]. The SCNS-SF34 has high internal consistency with Cronbach’s alpha of at least 0.86 for each subscale, and is moderately correlated with the Hospital Anxiety and Depression Scale, Distress Thermometer and Quality of Life Questionnaire-Core 30 (QLQ-C30) [27]. Furthermore, cancer patients have reported a preference for the SCNS-SF34 over the QLQ-C30, Functional Assessment of Cancer Therapy-General and Kingston Needs Assessment–Cancer as a strategy for conveying their needs to health care providers [29].

### Study factors

#### Individual

Age at diagnosis and sex were obtained from the cancer registry. Current marital status, highest level of education completed, health insurance coverage, current employment situation, geographical location, size of household, and presence of physical co-morbidities were obtained by questionnaire.

#### Disease and treatment

Primary cancer type and spread of disease at diagnosis were obtained from the cancer registry, with survivors’ cancer categorised as ‘early/less progressed’ (in-situ or localised; grade 1 or 2; T1 or T2), ‘late/more progressed’ (invasion of adjacent organs, regional nodes or distant metastases; grade 3 or 4; not T1) or ‘not applicable’ (haematological cancers). Extent of disease at six months post-diagnosis, and cancer treatments received in the last month were obtained by questionnaire.

#### Health behaviours

Seven questionnaire items adapted from existing measures assessed health behaviors: two items assessed smoking behavior, with participants classified as ‘current’, ‘former’ or ‘never smoker’ [30]; two items assessed alcohol consumption [31] and participants who consumed more than two standard drinks on any day were classified as being at ‘increased lifetime risk of harm’ from alcohol related injury or disease [32]; and three items assessed physical activity [33] with participants classified as ‘sufficiently active’ (at least 150 minutes of physical activity per week), ‘insufficiently active’ (participating in some activity but not enough in total time) or ‘sedentary’ (no physical activity) [34].

#### Psychological

Two questionnaire items assessed treatment for mental health illness (eg. depression, anxiety, schizophrenia) before and since the cancer diagnosis. Coping was assessed by the 21-item Mini Mental Adjustment to Cancer Scale (Mini-MAC) which measures five cancer-specific coping strategies: helplessness-hopelessness, anxious preoccupation, fighting spirit, cognitive avoidance and fatalism [35]. The mini-MAC has demonstrated reliability with Cronbach alpha coefficients for each subscale ranging from 0.62–0.88. Raw scores for each subscale were standardised from 0 to 100 [35] and survivors who scored in the top 16% of each distribution were classified as a ‘case’ on that specific coping strategy [36].

#### Social

Social support was assessed by the MOS Social Support Survey (MOS − SSS) which measures four domains of functional support: emotional/informational, tangible, affectionate, and positive social interaction [37]. Raw subscale scores were standardised from 0 to 100 and survivors who scored in the bottom one-third of each distribution were classified as having ‘low’ availability of that particular type of social support (Sherbourne, personal communication). The survey has high internal consistency with alpha coefficients exceeding 0.91 for each subscale and demonstrated validity with the chronic illness population [37].

### Statistical methods

Due to small numbers, data from survivors diagnosed with non-Hodgkin’s lymphoma or leukaemia were combined and categorised as ‘haematological’ cancer. The proportion of survivors who reported either ‘no needs’ (ie. selected response option 1 or 2 to all 34 items), ‘low needs’ (ie. selected response option 3 to at least one item, but did not select response option 4 or 5 to any items) and ‘moderate to high needs’ (ie. selected response option 4 or 5 to at least one item) was calculated overall and by cancer type, with 95% confidence intervals. The association between cancer type with reporting ‘no needs’, ‘low needs’ and ‘moderate to high needs’ was examined using chi-square analyses. For each domain, the proportion of survivors who reported ‘moderate to high needs’ versus ‘low or no needs’ was calculated with 95% confidence intervals. The proportion of survivors who endorsed each SCNS-SF34 item at either a ‘moderate’ or ‘high’ level was calculated with 95% confidence intervals and the ten most prevalent items and their corresponding domain identified. Chi-square analyses examined the association between survivors’ individual, disease, health behaviour, psychological and social characteristics with ‘moderate to high needs’ versus ‘low or no needs’ for each domain. Multiple logistic regression analyses were then conducted to examine factors associated with ‘moderate to high needs’ while adjusting for potential confounders. Variables with a p-value ≤0.2 on univariate analyses were included in a backward logistic regression model for each domain. Variables were removed from the model if they had a p-value <0.1 on the likelihood ratio test; those with a p-value ≤0.05 were considered statistically significant.

### Sample size

The registries were required to recruit a quota of 1660 eligible survivors who consented to being contacted about the study. Based on previous experience [6], we estimated that 80% of survivors would return a completed survey, resulting in a sample size of approximately 1320 at T1. Assuming a prevalence of moderate to high needs of 20%, a sample of this size would allow the proportion of survivors with unmet needs to be estimated with 95% confidence intervals within ± 3%, and provide 90% power to detect differences of 7% between categories of study factors associated with moderate to high needs at the 5% significance level.

## Results

### Sample

Of the 3877 potential participants assessed for study eligibility, 3315 were deemed eligible and of these, 1691 (51%) consented to being contacted about the study by the researchers. A total of 1360 eligible survivors returned a T1 survey (41% response rate at T1). Thirty seven participants who returned their T1 survey more than 9 months after diagnosis were excluded from analyses. The 1323 survivors included in these analyses were surveyed at a median of 6 months after diagnosis (SD = 1 month, minimum 4 months, maximum 9 months) and their median age was 63 years (SD = 11 years; minimum 18 years, maximum 80 years). More than half of the participants (59%) were male, about half were diagnosed with early stage disease (52%), the most common diagnosis was prostate cancer (26%), almost two-thirds (62%) were in remission at the time of survey completion and 72% had not received any active treatment in the last month. While the study sample reflected the national profile [24] for the top eight incident cancers diagnosed in 2005 in terms of gender and age, survivors of colorectal cancer appeared to be under-represented and haematological and head and neck cancers over-represented. Participant characteristics have been reported in detail elsewhere [22].

### Prevalence of supportive care needs

As shown in Table 1, 496 (42%, 95% CI: 39%-45%) survivors reported ‘no need’ for help with all of the 34 items assessed. A total of 444 (37%; 95% CI: 34%-40%) survivors reported having at least one ‘moderate to high’ level unmet supportive care need and of these, 53% (n = 237) had one to four ‘moderate to high’ needs and 47% (n = 207) had five or more ‘moderate to high’ needs. There was significant variation across cancer types in the percentage of survivors who reported unmet needs (χ^2^ = 91.39; df = 12; p < 0.001). ‘Moderate to high’ level unmet needs were most common amongst survivors of lung cancer with more than half (60%; 95% CI: 51%-69%) endorsing at least one item. Conversely, almost two-thirds (65%; 95% CI: 58%-72%) of melanoma survivors reported ‘no need’ for help with all items.

**Table 1:** Prevalence of supportive care needs at six months post-diagnosis by cancer type

	Total* (N = 1187)	**Prostate (n = 309)**	**Melanoma (n = 188)**	**Breast (n = 186)**	**Blood (n = 164)**	**Colorectal (n = 145)**	**Lung (n = 108)**	**Head & neck (n = 87)**
	**n % (95% CI)**	**n % (95% CI)**	**n % (95% CI)**	**n % (95% CI)**	**n % (95% CI)**	**n % (95% CI)**	**n % (95% CI)**	**n % (95% CI)**
No needs^†^	496	134	122	56	54	66	29	35
	42 (39–45)	43 (37–49)	65 (58–72)	30 (23–37)	33 (26–40)	46 (38–54)	27 (19–35)	40 (30–50)
Low needs^‡^	247	58	33	47	38	31	14	26
	21 (19–23)	19 (15–23)	17 (12–22)	25 (19–31)	23 (17–29)	21 (14–28)	13 (7–19)	30 (20–40)
Moderate to high needs^§^	444	117	33	83	72	48	65	26
	37 (34–40)	38 (33–43)	17 (12–22)	45 (38–52)	44 (36–52)	33 (25–41)	60 (51–69)	30 (20–40)

* includes those with no missing items across all domains.

^†^ selected ‘no’ need for help to all 34 items.

^‡^ selected ‘low’ level need for help to at least one item, but did not select ‘moderate’ or ‘high’ need to any item.

^§^ selected ‘moderate’ or ‘high’ level need for help to at least one item.

At the domain level, 318 (25%; 95% CI: 23%-27%) survivors reported unmet psychological needs, 251 (20%; 95% CI: 18%-22%) reported unmet physical aspects of daily living needs, and 232 (18%; 95% CI: 16%-20%) reported unmet health systems and information needs at a ‘moderate to high’ level. Only 167 (13%; 95% CI: 11%-15%) and 103 (8%; 95% CI: 7%-9%) survivors respectively reported ‘moderate to high’ level unmet need for help with sexuality, and patient care and support domains.

### Most prevalent ‘moderate to high’ level unmet supportive care needs

The 10 highest ranked items that survivors reported a ‘moderate to high’ level of need for help with are shown in Table 2. Overall, individual items were endorsed by relatively few (≤15%) survivors. The highest ranked items were *concerns about the worries of those close to you* (15%), *fears about the cancer spreading* (14%), *not being able to do the things they used to do* (13%), *uncertainty about the future* (13%), and *lack of energy/tiredness* (12%). Half of the top 10 needs items were from the psychological domain, three were from the physical aspects of daily living domain and the remaining two items were from the sexuality domain.

**Table 2:** Ten most prevalent ‘moderate’ or ‘high’ level unmet supportive care needs

**Rank**	**SCNS-SF34 item**	**Number (%) moderate or high needs**	**Domain**
1	Concerns about the worries of those close to you	192 (15)	Psychological
2	Fears about the cancer spreading	185 (14)	Psychological
3	Not being able to do the things you used to do	169 (13)	Physical/ daily living
4	Uncertainty about the future	168 (13)	Psychological
5	Lack of energy/tiredness	157 (12)	Physical/ daily living
6	Changes in your sexual relationships	140 (11)	Sexuality
7	Changes in sexual feelings	139 (11)	Sexuality
8	Work around the home	137 (11)	Physical/ daily living
9.	Worry that the results of treatment are beyond your control	128 (10)	Psychological
10	Feeling down or depressed	120 (9)	Psychological

Total number of observations for each item ranges from 1292–1302 due to missing values.

### Factors associated with ‘moderate to high’ level unmet need

The individual, health behaviour, disease, treatment, psychological and social characteristics associated with survivors reporting ‘moderate to high’ level unmet needs by domain are shown in Tables 3, 4 and 5. Domains are displayed side-by-side for ease of comparison.

**Table 3:** Individual and health behaviour characteristics associated with moderate to high level unmet needs by domain*

	**Psychological**	**Physical & daily living**	**Sexuality**	**Health system & information**	**Patient care & support**
	***p-value*Adjusted OR (95% CI)**	***p-value*Adjusted OR (95% CI)**	***p-value*Adjusted OR (95% CI)**	***p-value*Adjusted OR (95% CI)**	***p-value*Adjusted OR (95% CI)**
**Individual**					
*Sex*			*0.089*		
Female			0.56 (0.29-1.1)		
Male			1.00		
*Marital status*			*<0.001*		
Married/ defacto			3.0 (1.6-5.6)		
Single/ widowed			1.00		
*Age at diagnosis*	*0.06*		*0.002*	*0.008*	
49 and younger	1.7 (0.98-2.8)		4.4 (1.8-10.6)	2.9 (1.5-5.5)	
50-59	1.1 (0.67-1.8)		4.3 (2.0-9.1)	2.5 (1.3-4.6)	
60-69	0.88 (0.55-1.4)		2.7 (1.5-5.2)	2.2 (1.3-3.9)	
70 and older	1.00		1.00	1.00	
*Current employment*		*<0.001*	*0.005*		
Paid work		0.78 (0.51-1.2)	0.92 (0.54-1.6)		
Not working		1.8 (1.2-2.8)	2.0 (1.1-3.4)		
Retired		1.00	1.00		
**Health behaviour**					
*Physical activity*	*0.05*	*<0.001*			
Sedentary	1.7 (1.1-2.7)	2.5 (1.6-4.0)			
Insufficient	1.5 (0.99-2.1)	1.8 (1.2-2.7)			
Sufficient	1.00	1.00			

* also adjusted for disease, treatment, psychological and social characteristics as reported in Tables 4 and 5.

OR = odds ratio; CI = confidence interval; p-value on the Wald chi-square analysis of effects test.

**Table 4:** Disease and treatment characteristics associated with moderate to high level unmet needs by domain*

	**Psychological**	**Physical & daily living**	**Sexuality**	**Health system & information**	**Patient care & support**
	***p-value*Adjusted OR (95% CI)**	***p-value*Adjusted OR (95% CI)**	***p-value*Adjusted OR (95% CI)**	***p-value*Adjusted OR (95% CI)**	***p-value*Adjusted OR (95% CI)**
**Disease**					
*Cancer status*				*<0.001*	*0.001*
Not remission				2.0 (1.4-2.9)	2.2 (1.4-3.5)
Remission				1.00	1.00
*Cancer type*		*0.003*	*<0.001*		
Breast		2.3 (1.1-4.6)	9.0 (2.5-32.2)		
Colorectal		2.1 (0.97-4.5)	6.4 (1.7-24.3)		
Blood		2.2 (1.1-4.5)	4.3 (1.2-15.5)		
Head neck		1.0 (0.41-2.5)	1.1 (0.21-6.1)		
Lung		4.1 (2.0-8.7)	5.8 (1.6-21.8)		
Prostate		1.7 (0.86-3.4)	23.1 (6.7-80.4)		
Melanoma		1.00	1.00		
**Treatment**					
*Surgery*			*0.093*		
Yes			2.1 (0.89-4.8)		
No/DK			1.00		
*Chemotherapy*	*0.005*	*0.023*			
Yes	1.8 (1.2-2.8)	1.6 (1.1-2.5)			
No/DK	1.00	1.00			
*Radiotherapy*	*0.05*				
Yes	1.6 (0.99-2.7)				
No/DK	1.00				
*Other*				*0.062*	
Yes				2.3 (0.96-5.7)	
No/DK				1.00	

* also adjusted for individual, health behaviour, psychological and social characteristics as reported in Tables 3 and 5.

OR = odds ratio; CI = confidence interval; p-value on the Wald chi-square analysis of effects test; DK = don’t know.

**Table 5:** Psychological and social characteristics associated with moderate to high level unmet needs by domain*

	**Psychological**	**Physical & daily living**	**Sexuality**	**Health system & information**	**Patient care & support**
	***p-value*Adjusted OR (95% CI)**	***p-value*Adjusted OR (95% CI)**	***p-value*Adjusted OR (95% CI)**	***p-value*Adjusted OR (95% CI)**	***p-value*Adjusted OR (95% CI)**
**Psychological**					
*Anxious preoccupation*	*<0.001*	*<0.001*	*<0.001*	*<0.001*	*<0.001*
Case	5.9 (4.0-8.7)	2.2 (1.5-3.2)	3.4 (2.2-5.3)	3.3 (2.2-5.1)	3.1 (1.8-5.2)
No case	1.00	1.00	1.00	1.00	1.00
*Helpless hopeless*	*<0.001*			*0.016*	*0.002*
Case	2.2 (1.5-3.3)			1.7 (1.1-2.7)	2.3 (1.3-3.8)
No case	1.00			1.00	1.00
*Cognitive avoidance*	*0.02*		*0.049*		
Case	1.5 (1.1-2.1)		1.5 (1.0-2.3)		
No case	1.00		1.00		
*Mental health help before cancer*		*<0.001*	*0.032*		*<0.001*
Yes		2.1 (1.4-3.2)	1.7 (1.0-2.8)		2.5 (1.5-4.2)
No		1.00	1.00		1.00
*Mental health help since cancer*	*<0.001*				
Yes	2.9 (1.6-5.2)				
No	1.00				
**Social**					
*Affectionate support*				*0.020*	*0.003*
Low				0.47 (0.25-0.89)	2.1 (1.3-3.4)
Some				1.00	1.00
*Positive social interaction*		*0.05*	*0.014*	*0.002*	
Low		1.4 (0.99-2.0)	1.7 (1.1-2.5)	2.6 (1.4-4.8)	
Some		1.00	1.00	1.00	
*Emotional/ informational*	*0.04*			*0.002*	
Low	1.4 (1.0-2.1)			2.2 (1.3-3.6)	
Some	1.00			1.00	

* also adjusted for individual, health behaviour, disease and treatment characteristics as reported in Tables 3 and 4.

OR = odds ratio; CI = confidence interval; p-value on the Wald chi-square analysis of effects test.

#### Individual

Age at diagnosis and current employment status were associated with multiple domains of unmet need (see Table 3). The odds of reporting sexuality, and health system and information needs increased with decreasing age. Compared to those who were retired, survivors who were currently not working (on leave, student, unemployed) or doing unpaid work (volunteer, household duties) had about twice the odds of reporting physical aspects of daily living and sexuality needs as those who were retired. Age was marginally non-significantly associated with psychological need. Married or defacto survivors had three times the odds of unmet sexuality needs compared to those who were single or widowed.

#### Health behaviour

Physical activity was the only health behaviour associated with moderate to high level unmet needs (see Table 3). The odds of reporting unmet psychological, and physical and daily living needs increased with decreasing levels of physical activity.

#### Disease and treatment

Cancer status, cancer type and having received chemotherapy in the last month were associated with multiple domains of unmet need (see Table 4). Compared to survivors in remission, those not in remission (stable, recurrent, metastatic disease) had about twice the odds of unmet health system and information, and patient care and support needs. Compared with survivors of melanoma, survivors of all other cancer types except head and neck had at least four times the odds of unmet sexuality needs, while lung, breast and haematological cancer survivors had at least two times the odds of unmet physical and daily living needs. Survivors who received chemotherapy in the last month had higher odds of unmet psychological, and physical and daily living needs than those who didn’t receive chemotherapy in the last month.

#### Psychological

Coping strategy and mental health treatment were associated with multiple domains of unmet need (see Table 5). Survivors who engaged in anxious preoccupation coping had two to six times higher odds of reporting unmet needs across all domains compared to survivors who did not use this coping strategy. Survivors who used helpless-hopeless coping had about twice the odds of reporting unmet psychological, health system and information, and patient care and support needs compared to those who didn’t use this strategy, while those who used cognitive avoidance coping had higher odds of reporting unmet psychological needs compared to those who didn’t use this strategy. Compared to survivors without a history of mental health treatment, those who had been treated for such problems before their cancer diagnosis had around twice the odds of unmet physical and daily living, and patient care and support needs, while those who had been treated for such problems since their cancer diagnosis had almost three times higher odds of unmet psychological needs.

#### Social

Compared to those with some affectionate support, survivors who perceived they had low levels of affectionate support had lower odds of health system and information, and higher odds of patient care and support needs. Compared to survivors with some positive social interaction, survivors who perceived that they had low levels of positive social interaction had higher odds of unmet sexuality, and health system and information needs. Survivors who perceived low levels of emotional/informational support also had higher odds of unmet health system and information needs (see Table 5).

## Discussion

This study found that six months after a cancer diagnosis, about one-third (37%) of survivors reported one or more items of moderate or high level unmet need, while almost two thirds (63%) reported either no or low level unmet needs. The most commonly reported moderate to high level unmet needs were from the psychological and physical and daily living domains. This is consistent with other recent needs assessments conducted with samples of cancer survivors at the end of treatment [17], in early phases of survivorship [15,18] and in long-term survivorship [14,16]. However, previous studies [13,15,17,18] found between 43%-60% of survivors reported at least one moderate or high level unmet need, compared to 37% of survivors in this study. Similarly, unlike earlier studies which found the most prevalent item of moderate or high unmet need occurred among 27-40% of recent survivors [13,17,18], the most commonly reported item of unmet need in this study was endorsed by only 15% of survivors.

The prevalence of unmet need reported by survivors in this study is clearly lower than previously reported, despite using the same validated instrument, and classification of unmet need. This may be because earlier studies of cancer survivors diagnosed with a diversity of cancer sites did not use population-based samples [13,17,18] and are therefore more susceptible to selection bias. In contrast, we used the two largest state-based cancer registries in Australia to assemble a population-based sample of survivors in the very early stages of cancer survivorship. Given that the study sample is generally representative of the source population, we are confident in our findings that most survivors’ supportive care needs, as measured by the SCNS-SF34, are relatively well met.

Due to the size and composition of the study sample, we were able to directly compare the prevalence of supportive care needs between seven common cancer types in Australia [26]. This bivariable analysis revealed significant variation across cancer types, with particularly low levels of unmet need reported by survivors of melanoma, 65% of whom reported no items of unmet need. This is fitting with our anecdotal experience whereby participants who were survivors of melanoma often questioned the legitimacy of their contribution to the study as they perceived themselves to have suffered less than survivors of other cancer types, and therefore less deserving of attention. Australia has the world’s highest incidence rate of melanoma; it is typically identified at early stages when simple treatment such as surgery will achieve a good prognosis [38]. It is possible that the omission of melanoma survivors from the sample composition of previous studies [17,18] may have contributed to their higher prevalence of unmet need compared to this study. In contrast, the highest levels of unmet need were reported by survivors of lung cancer, with 60% reporting at least one item of moderate or high level need. Given the high level of burden associated with lung cancer in terms of poor prognosis, treatment side effects and declining physical health, this finding is not surprising.

Subgroups of survivors with domain-specific and widespread unmet needs were identified. After adjusting for a comprehensive range of individual, disease, health behaviour, psychological and social factors, cancer type was found to be significantly associated with moderate to high level unmet physical and daily living, and sexuality needs only. In particular, survivors of lung cancer had the highest odds of reporting unmet physical and daily living needs, while survivors of prostate cancer had extremely high odds of reporting unmet sexuality needs. These findings suggest that the type of unmet need experienced by survivors does not routinely differ between cancer types. Rather, the notion of cancer site- specific unmet needs appears to apply to only a few explicit dimensions of unmet need.

Consistent with previous studies, not being in remission was associated with unmet health system and information, and patient care and support needs; this is not surprising given this subgroup of survivors is likely to be receiving intermittent treatment and symptom management. While almost three quarters of survivors reported not receiving any active treatment in the last month, we did not assess if participants had completed all active treatments given the changeable and uncertain nature of adjuvant treatment regimes. While each treatment was considered separately, having received chemotherapy in the last month was the only treatment associated with higher odds of reporting unmet needs. Interestingly, physical activity was the only health behaviour associated with unmet needs, with sedentary survivors reporting higher odds of unmet psychological, and physical and daily living needs. Although 37% of the sample resided in regional or remote areas, our results did not support the findings from previous studies of an association between rural location and unmet needs. On account of the range of study factors examined in this study, a number of associations were established for the first time. Low levels of social support and maladaptive coping styles were associated with multiple domains of unmet need. Notably, survivors who were identified as a case on anxious preoccupation coping had more than twice the odds of reporting unmet needs across all five domains. While causation cannot be inferred, the new associations identified in this study are particularly valuable because social support and coping style are potentially amenable to intervention. In particular, attention could be directed towards exploring the contribution that targeted coping interventions focusing on anxiety and helplessness, could make towards the prevention of or reduction in survivors’ unmet needs across a number of domains.

### Strengths and limitations

While previous needs assessments have also included a diversity of recent cancer survivors [13,17,18], the population-based sampling method used in this study is a major strength as it increases the generalisability of the results. In Australia, the notification of cancer to the cancer registry is a statutory requirement under the state and territory Public Health Acts. Indices of registry data quality demonstrate that the level of case ascertainment is high and the data collected are accurate [39]. However, the overall response rate was 41% (1360/3315 eligible individuals) and may raise concerns about response bias. While this response rate seems low, it is higher than that achieved by other studies which also used cancer registries to recruit diverse samples of recent survivors [40,41]. Survivors who were not proficient in English were excluded due to the prohibitive cost of translating the questionnaire into other languages and may have resulted in an underestimate of the prevalence of unmet needs given that language barriers have been associated with poorer access to health care services. Our outcome measure, the SCNS-SF34, is a well-validated tool for assessing multiple dimensions of supportive care need and was developed with diverse samples of individuals diagnosed with cancer in terms of cancer type and time since diagnosis [27]. However, it is possible that the SCNS-SF34 may not fully capture the unique needs of cancer survivors in the late treatment to early survivorship phase of care, and therefore this study may underestimate the prevalence of unmet need reported by survivors at six months post-diagnosis. Since this study commenced, two cancer survivor-specific needs assessment tools [41,42] have been developed and should be considered for use in future studies.

## Conclusions

About one-third of cancer survivors in the transition from late treatment to early survivorship had moderate to high levels of unmet need, particularly in the psychological and daily living domains. Our findings directly inform health care professionals and organisations involved in the provision of survivorship care about the actions, resources and services most needed by subgroups of survivors. Our findings also suggest that coping support interventions may have the potential to contribute to the prevention or reduction of survivors’ unmet needs across all domains. However, it is important not to overlook the finding that 63% of survivors in this study reported no or low level unmet needs at six months post-diagnosis and for whom current care appears to adequately meet their needs. On the basis that a valuable new perspective about how to prevent or reduce cancer survivors’ unmet needs could be gained from those with no unmet needs, future research should seek to identify and better understand this subgroup of survivors.

## Competing interests

The authors declare that they have no competing interests.

## Authors’ contributions

AB participated in study conception, design and acquisition of funding; was responsible for implementing the study protocol; performed some of the statistical analysis; interpreted the data and drafted the manuscript. AG participated in study conception, design and acquisition of funding. CD participated in the study design and acquisition of funding, and coordinated the statistical analysis. AZ helped to implement the study protocol and performed some of the statistical analysis. All authors participated in revising the manuscript, and read and approved the final version.

## Pre-publication history

The pre-publication history for this paper can be accessed here:

http://www.biomedcentral.com/1471-2407/12/150/prepub

## References

[B1] Ferlay J Shin HR Bray F Forman D Mathers C Parkin DM GLOBOCAN 2008, Cancer Incidence and Mortality Worldwide: IARC CancerBase No 10 [http://globocan.iarc.fr/]

[B2] Zucca AC Boyes AW Linden W Girgis A All’s well that ends well? Quality of life and physical symptom clusters in long-term cancer survivors across cancer types J Pain Symptom Manage 2012 43 720 731 10.1016/j.jpainsymman.2011.04.023 22277904

[B3] Burton AW Fanciullo GJ Beasley RD Fisch MJ Chronic pain in the cancer survivor: a new frontier Pain Med 2007 8 189 198 10.1111/j.1526-4637.2006.00220.x 17305690

[B4] Kuchinski AM Reading M Lash AA Treatment-related fatigue and exercise in patients with cancer: a systematic review Medsurg Nurs 2009 18 174 180 19591364

[B5] Deimling GT Bowman KF Sterns S Wagner LJ Kahana B Cancer-related health worries and psychological distress among older adult, long-term cancer survivors Psychooncology 2006 15 306 320 10.1002/pon.955 16041841

[B6] Boyes A Girgis A Zucca A Lecathelinais C Anxiety and depression among long-term survivors of cancer in Australia: results of a population-based survey Med J Aust 2009 190 Suppl 7 S94 S98 19351302 10.5694/j.1326-5377.2009.tb02479.x

[B7] Short PF Vasey JJ Tunceli K Employment pathways in a large cohort of adult cancer survivors Cancer 2005 103 1292 1301 10.1002/cncr.20912 15700265

[B8] Foster C Wright D Hill H Psychosocial implications of living 5 years or more following a cancer diagnosis: a systematic review of the research evidence Eur J Cancer Care 2009 18 223 247 10.1111/j.1365-2354.2008.01001.x 19432917

[B9] Wen KY Gustafson DH Needs assessment for cancer patients and their families Health Qual Life Outcomes 2004 2 11 10.1186/1477-7525-2-11 14987334 PMC394345

[B10] Gustafson DH Lipscomb J, Gotay CC, Snyder CF Needs assessment in cancer Outcomes assessment in cancer: Measures, methods and applications 2005 Cambridge University Press, Cambridge 305 328

[B11] Richardson A Medina J Brown V Sitzia J Patients’ needs assessment in cancer care: a review of assessment tools Support Care Cancer 2007 15 125 1144 10.1007/s00520-006-0205-8 17235503

[B12] Harrison JD Young JM Price MA Butow PN Solomon MJ What are the unmet supportive care needs of people with cancer? a systematic review Support Care Cancer 2009 17 1117 1128 10.1007/s00520-009-0615-5 19319577

[B13] Sanson-Fisher R Girgis A Boyes A Bonevski B Burton L Cook P Supportive Care Review Group The unmet supportive care needs of patients with cancer Cancer 2000 88 226 237 10.1002/(SICI)1097-0142(20000101)88:1<226::AID-CNCR30>3.0.CO;2-P 10618627

[B14] Hodgkinson K Butow P Fuchs A Hunt GE Stenlake A Hobbs KM Brand A Wain G Long-term survival from gynecologic cancer: Psychosocial outcomes, supportive care needs and positive outcomes Gynecol Oncol 2007 104 381 389 10.1016/j.ygyno.2006.08.036 17027072

[B15] Beesley V Eakin E Steginga S Aitken J Dunn J Battistutta D Unmet needs of gynaecological cancer survivors: implications for developing community support services Psychooncology 2008 17 392 400 10.1002/pon.1249 17680554

[B16] Hodgkinson K Butow P Hunt GE Pendlebury S Hobbs KM Wain G Breast cancer survivors' supportive care needs 2–10 years after diagnosis Support Care Cancer 2007 15 515 523 10.1007/s00520-006-0170-2 17120068

[B17] Armes J Crowe M Colbourne L Morgan H Murrells T Oakley C Palmer N Ream E Young A Richardson A Patients’ supportive care needs beyond the end of cancer treatment: a prospective, longitudinal survey J Clin Oncol 2009 27 6172 6179 10.1200/JCO.2009.22.5151 19884548

[B18] McDowell ME Occhipinti SS Ferguson M Chambers SK Predictors of change in unmet supportive care needs in cancer Psychooncology 2010 19 508 516 10.1002/pon.1604 19598292

[B19] Girgis A Boyes A Sanson-Fisher R Burrows S Perceived needs of women diagnosed with breast cancer: a focus on rural versus urban location Aust NZ J Public Health 2000 24 166 173 10.1111/j.1467-842X.2000.tb00137.x 10790936

[B20] Hewitt M Greenfield S Stovall E From cancer patient to cancer survivor: Lost in transition 2006 The National Academies Press, Washington DC

[B21] Brearley SG Stamataki Z Addington-Hall J Foster C Hodges L Jarrett N Richardson A Scott I Sharpe M Stark D Siller C Ziegler L Amir Z The physical and practical problems experienced by cancer survivors: a rapid review and synthesis of the literature Eur J Oncol Nurs 2011 15 204 212 10.1016/j.ejon.2011.02.005 21489873

[B22] Boyes AW Girgis A D’Este C Zucca AC Flourishing or floundering? Prevalence and correlates of anxiety and depression among a population-based sample of adult cancer survivors 6 months after diagnosis J Affect Disord 2011 135 184 192 10.1016/j.jad.2011.07.016 21864913

[B23] Hall AE Boyes AW Bowman J Walsh RA James EL Girgis A Young adult cancer survivors’ psychosocial well-being: a cross-sectional study assessing quality of life, unmet needs and health behaviors Support Care Cancerin press 10.1007/s00520-011-1221-x 21720746

[B24] Feuerstein M Defining cancer survivorship J Cancer Surviv 2007 1 5 7 10.1007/s11764-006-0002-x 18648939

[B25] Office of Cancer Survivorship About cancer survivorship – definitions [http://dccps.nci.nih.gov/ocs/definitions.html]

[B26] Australian Institute of Health and Welfare (AIHW) & Australasian Association of Cancer Registries (AACR) Cancer in Australia: an overview, 2008 2008 AIHW & AACR, Cancer series no 46. Canberra

[B27] Boyes A Girgis A Lecathelinais C Brief assessment of adult cancer patients’ perceived needs: development and validation of the 34-item Supportive Care Needs Survey (SCNS-SF34) J Eval Clin Pract 2009 15 602 606 10.1111/j.1365-2753.2008.01057.x 19522727

[B28] McElduff P Boyes A Zucca A Girgis A The Supportive Care Needs Survey: a guide to administration, scoring and analysis 2004 Centre for Health Research & Psycho-Oncology, Newcastle

[B29] Snyder CF Dy SM Hendricks DE Brahmer JR Carducci MA Wolff AC Asking the right questions: investigating needs assessments and health-related quality-of-life questionnaires for use in oncology clinical practice Support Care Cancer 2007 15 1075 1085 10.1007/s00520-007-0223-1 17318591

[B30] Australian Institute of Health and Welfare (AIHW) National Health Data Dictionary, Version 8.0 1999 AIHW, Canberra

[B31] Australian Institute of Health and Welfare (AIHW) 2001 National Drug Strategy Household Survey: Detailed findings 2002 AIHW, Canberra

[B32] National Health and Medical Research Council (NHMRC) Australian Guidelines: to reduce health risks from drinking alcohol 2009 NHMRC, Canberra

[B33] Australian Institute of Health and Welfare (AIHW) The Active Australia Survey: a guide and manual for implementation, analysis and reporting 2003 AIHW, Canberra

[B34] Australian Government Department of Health and Ageing National Physical Activity Guidelines for Australians 1999 AGPS, Canberra

[B35] Watson M Law M dos Santos M Greer S Baruch J Bliss JM The Mini-MAC: further development of the Mental Adjustment to Cancer Scale J Psychosoc Oncol 1994 12 33 46 10.1300/J077V12N03_03

[B36] Watson M Greer S Bliss J Mental Adjustment to Cancer (MAC) Scale Users Manual 1989 CRC Psychological Medicine Research Group, Surrey

[B37] Sherbourne C Stewart A The MOS Social Support Survey Soc Sci Med 1991 32 705 714 10.1016/0277-9536(91)90150-B 2035047

[B38] Australian Cancer Network Melanoma Guidelines Revision Working Party Clinical Practice Guidelines for the Management of Melanoma in Australia and New Zealand 2008 Cancer Council Australia and Australian Cancer Network, Wellington

[B39] Curado MP, Edwards B, Shin HR, Storm H, Ferlay J, Heanue M, Boyle P Cancer Incidence in Five Continents, Vol IX 2007 IARC, Lyon

[B40] Smith T Stein KD Mehta CC The rationale, design, and implementation of the American Cancer Society’s Studies of Cancer Survivors Cancer 2007 109 1 10 10.1002/cncr.22387 17146781

[B41] Campbell SH Sanson-Fisher RW Turner D Hayward L Wang S Taylor-Brown J Psychometric properties of Cancer Survivors' Unmet Needs Survey Support Care Cancer 2010 19 221 230 20099001 10.1007/s00520-009-0806-0

[B42] Hodgkinson K Butow P Hunt GE Pendlebury S Hobbs KM Lo SK Hunt GE Wain G The development and evaluation of a measure to assess cancer survivors’ unmet supportive care needs: the CaSUN (Cancer Survivors’ Unmet Needs measure) Psychooncology 2007 16 796 804 10.1002/pon.1137 17177268

